# Long-term social skills group training for children and adolescents with autism spectrum disorder: a randomized controlled trial

**DOI:** 10.1007/s00787-018-1161-9

**Published:** 2018-05-10

**Authors:** Ulf Jonsson, Nora Choque Olsson, Christina Coco, Anders Görling, Oskar Flygare, Anna Råde, Qi Chen, Steve Berggren, Kristiina Tammimies, Sven Bölte

**Affiliations:** 1 Pediatric Neuropsychiatry Unit, Department of Women’s and Children’s Health, Center of Neurodevelopmental Disorders at Karolinska Institutet (KIND), Karolinska Institutet, Gävlegatan 22B, SE-11330 Stockholm, Sweden; 2 Child and Adolescent Psychiatry, BUP-KIND, Center for Psychiatry Research, Stockholm County Council, Stockholm, Sweden; 3 Department of Neuroscience, Child and Adolescent Psychiatry, Uppsala University, Uppsala, Sweden; 4 Medical Epidemiology and Biostatistics, Karolinska Institutet, Stockholm, Sweden

**Keywords:** Autism, Neurodevelopmental disorder, Long-term, Social skills, Group training

## Abstract

Social skills group training (SSGT) is widely used for intellectually able children and adolescents with autism spectrum disorder (ASD). Previous studies indicate small to moderate effects on social communication capacities. The duration of most available programs is relatively short, and extended training might lead to further improvement. This randomized controlled trial compared an extended 24-week version of the SSGT program KONTAKT with standard care. The weekly sessions gradually shifted in content from acquisition of new skills to real-world application of the acquired skills. A total of 50 participants with ASD (15 females; 35 males) aged 8–17 years were included. The study was conducted at two child and adolescent psychiatry outpatient units in Sweden. The primary outcome was the Social Responsiveness Scale–Second Edition (SRS-2) rated by parents and blinded teachers. Secondary outcomes included parent- and teacher-rated adaptive behaviors, trainer-rated global functioning and clinical severity, and self-reported child and caregiver stress. Assessments were made at baseline, posttreatment, and at 3-months follow-up. Parent-rated SRS-2 scores indicated large effects posttreatment [− 19.2; 95% CI − 29.9 to − 8.5; *p* < .001, effect size (ES) = 0.76], which were maintained at follow-up (− 20.7; 95% CI − 31.7 to − 9.7; *p* < .0001, ES = 0.82). These estimates indicate substantially larger improvement than previously reported for shorter SSGT. However, the effects on teacher-rated SRS-2 and most secondary outcomes did not reach statistical significance. Our results suggest added benefits of extended SSGT training, implying that service providers might reach better results by optimizing the delivery of SSGT.

**Electronic supplementary material:**

The online version of this article (10.1007/s00787-018-1161-9) contains supplementary material, which is available to authorized users.

## Introduction

Persistent challenges in social communication and social interaction across multiple contexts are main characteristics of autism spectrum disorder (ASD) [1]. Effective interventions that address these difficulties are desirable, and should aim to provide autistic individuals with skills that enable them to gain improved self-confidence and control over their social lives. This could, in turn, prevent negative outcomes associated with ASD such as comorbid mental disorders [2], bullying [3], loneliness [4], and school absenteeism [5]. Despite widespread demand, however, access to evidence-based interventions for individuals with ASD remains limited.

Social skills group training (SSGT) is an umbrella term for interventions applying socially instructive techniques and behavioral modification principles in group settings to improve social skills, typically used in the clinical management of children and adolescents with ASD in the normative intellectual range. The interventions vary considerably in their content and structure. A recent systematic review, including 18 trials comprising a total of 745 participants, indicated small to moderate effect sizes for parent- and observer-report (*g* = 0.47 and 0.40, respectively) and non-significant effects for teacher-report [6]. Two subsequently published large-scale randomized controlled trials (RCTs) also suggested that the gains in terms of social skills are limited [7, 8]. The first of these trials was a multi-center trial performed at six German universities with specialized ASD outpatient clinics, in which 228 children and adolescents were randomized to 12 sessions of a group-based cognitive behavioral program or treatment as usual. This study reported a small effect size (*d* = 0.33) three months after the intervention ended [8]. The second trial, which is the largest to date, was a multi-center trial conducted at 13 child and adolescent psychiatry outpatient units in Sweden and coordinated by our research center. A total of 296 children and adolescents were randomized to a 12-week version of the SSGT ‘KONTAKT’ or standard care only. The effect, as measured by the parent-rated Social Responsiveness Scale–Second Edition (SRS-2) [9] three months after completed training, was small (*d* = 0.16) and only statistically significant for the adolescent subsample (*d* = 0.33) and for girls (*d* = 0.40) [7].

The scientific evidence base for SSGT in ASD has evolved from initial small pilot trials conducted in university settings to more recent pragmatic multi-center trials performed in real-world clinical settings. Despite this progress, there are still major gaps in the literature. For instance, the SSGT interventions have rarely been tested against active comparators, outcome measures are often unblinded, and the key components and mechanisms of change are poorly understood [6, 10]. Specially, one important and largely unanswered issue is to what extent more training provides additional benefits [6]. By understanding the potential benefits of longer periods of training, service providers would be able to optimize the training in terms of both costs and effects. The duration of most SSGT programs investigated in previous studies was typically no more than 3–4 months. A few lasted only 4–5 weeks, while two programs lasted as long as 20 weeks [10]. Even though the intensity varied across different programs, their relatively short duration is in stark contrast to many other common interventions for individuals with ASD, such as early intensive behavioral intervention for young children with ASD which typically involves many hours of training per week over years. Added benefits from longer periods of training would certainly make sense from a theoretical perspective. Prolonged periods of focused practice are generally necessary for humans to acquire and maintain complex skills [11]. It is unlikely that social skills are exceptions. While such skills are acquired implicitly in typical development, individuals with ASD may need to learn them explicitly. Still, with enduring practice a transition from explicit to implicit processing might occur [12], making the performance of the new skills more natural and effective over time. In particular, individuals with learning challenges in certain areas are likely to benefit from programs that allow for overlearning so that a skill can slowly become an integrated part of their repertoire [13]. On the other hand, more intense interventions might increase the risk of fatigue, refusal, and drop-out. It should also be noted that long-term training does not necessarily mean more of the same. In particular, longer period of training allows for a gradual shift in content from the acquisition of new skills towards the application of these skills in situations of relevance for the participants’ everyday lives. Additionally, an incremental increase in the tailoring of the intervention for each unique participant is possible.

This study aimed to estimate the effects of a longer version of SSGT KONTAKT in children and adolescents with ASD. The program consisted of 24 weekly sessions, with a gradual increase in tailoring and focus on complex skills in real-world situations. We hypothesized that children and adolescents with ASD who received the program would show increased social communication skills and improved daily adaptation compared to a control group receiving standard care only. We also hypothesized that the intervention would reduce perceived stress, general symptom severity, and increase the global level of functioning.

## Method

### Study design

This was a 24-week RCT evaluating the effect of an extended version of KONTAKT as a complement to standard care, compared with standard care only. The study was conducted at two units within the regional child and adolescent mental health service in Stockholm, Sweden, between March 2013 and September 2015. To reflect clients typically referred to real-world clinical services, a sample of children and adolescents with ASD showing diverse psychiatric comorbidity was recruited. The study was conducted in parallel to the previously published evaluation of the shorter 12-week version of KONTAKT [7]. The designs and procedures of these trials were largely identical as regards outcome measures, recruitment strategy, and training and supervision of the staff delivering the intervention. The studies had three points of assessment: baseline, posttreatment, and 3-months follow-up. As a consequence of the difference in duration, the posttreatment and follow-up assessment occurred 12 weeks later in the present trial, which precludes direct comparisons of the two trials. An additional difference was that the present trial was conducted at only two of the 13 clinical units where the shorter version was implemented. Coordinating activities, data management and analysis were conducted at the Center of Neurodevelopmental Disorders at Karolinska Institutet, KIND. The study was approved by the Ethical Review Board in Stockholm (2012/385-31/4) and is registered with ClinicalTrials.gov [identifier: NCT01854346].

### Participants

Eligible participants were children and adolescents (7–17 years) with a diagnosis of ASD (F84.0, F84.1, F84.5, or F84.9) according to the International Classification of Diseases, 10th Revision (ICD-10) [14] established by multidisciplinary assessment teams in regular healthcare services [15], and corroborated by met ASD cut-offs (modules 3 or 4) on the Autism Diagnostic Observation Schedule (ADOS) [16] conducted by certified examiners. Eligible participants also had a previously established diagnosis of ADHD (F90.0 or F90.8), anxiety disorder (F40, F41 or F43), or depression (F32 or F33), corroborated by the Kiddie-Schedule of Affective Disorders and Schizophrenia (K-SADS) [17] in case of uncertainty. They also had an IQ > 70 determined through the Wechsler Intelligence Scale for Children-third or fourth edition (WISC-III/IV) [18, 19]. Exclusion criteria were a history of clinically assessed self-injury, conduct disorder (F91), hyperkinetic conduct disorder (F90.1), antisocial personality disorder (F60.2), borderline personality disorder (F60.3), any form of schizophrenia or related psychotic disorder (F20–F29) that would interfere with participation or require alternative treatment, and insufficient Swedish language capacities.

### Procedure

The participants were recruited by either self-referral or by referral from mental health services. After a preliminary telephone screening interview, an extended interview was conducted to verify each applicant’s eligibility. Relevant medical records were checked to verify reported diagnoses and to collect available results from WISC-III/-IV and ADOS assessments. If necessary, supplementary assessments were performed. The participants received a 100 SEK (11 EUR) voucher as an incentive for participating in the trial. Written consent was obtained from each participant and/or parent/guardian after the study’s aims and procedures had been fully explained. The assessments comprised questionnaires for participants, parents, teachers and clinicians and interviews with the participants and their parents. The trial was conducted at one child and adolescent psychiatry outpatient unit (BUP-Brommaplan) and one academic clinical outpatient unit (BUP-KIND) in Stockholm. Seven licensed psychologists with a mean 3.75 years (SD = 3.47) of experience in working with ASD conducted the training. Trainers in the present trial and the simultaneously performed evaluation of the short version of KONTAKT were systematically trained on the program, including theory, supervision, and feedback on recorded sessions [7]. Adherence to protocol was maintained by giving trainers continuous supervision throughout the trial (monthly 3-h sessions during the first 12 weeks, and a 1-h session every second week the following 12 weeks). Adherence was verified by screening random samples of video-recoded sessions, using a checklist containing 11 items regarding protocol adherence and trainer skills in applying basic principles of the SSGT (e.g., positive reinforcement, modeling, prompting). Each item was scored “0” for no adherence, “1” for some adherence, and “2” for full adherence, and a mean score was derived across the items. Twenty-seven video recordings were screened. With a mean rating of 1.65 (SD = 0.30), the overall treatment fidelity was deemed satisfactory.

### Randomization and masking

The randomization was performed by a senior researcher using computer-generated random numbers (http://www.random.org) stratified by age groups (children aged 7–12 and adolescents aged 13–17). Participants in each group were randomly assigned to the experimental or control group using block randomization in a 1:1 ratio. Parents and trainers were aware of the treatment conditions, while teachers were blinded to treatment conditions. The latter was ensured by a teacher survey showing no awareness of group allocation beyond chance.

### Intervention

KONTAKT is a manualized and structured group training for children and adolescents with ASD in the normative IQ range, developed in Germany [20] and adapted for Swedish conditions [21, 22]. It aims to improve social interaction and communication skills, social motivation, awareness of self and others, problem-solving capacities, and self-confidence. The program applies elements of cognitive behavioral therapy, computer-based cognitive training, behavior activation, psychoeducation, observational learning, and parent involvement applying various mandatory, recurring, and variable treatment formats. Training sessions focus on understanding social rules and relationships, initiating social overtures, developing conversation skills, identifying and interpreting verbal and non-verbal social signals, managing conflicts, and developing social communication coping strategies. The teaching formats include individual goal identification, group discussions, social and role play, emotion-processing training, group activities, and homework assignments. Children are trained for 60 min. and adolescents for 90 min. per week in groups of 4–8 participants with 2–3 trainers. The Swedish version of KONTAKT has a supplementary workbook for group leaders, participants, and parents with additional information about content for each session of the treatment to enhance clinical feasibility, treatment integrity and adherence. There are two versions of KONTAKT: a short version (12 weekly sessions), and an extended version (24 weekly sessions). As mentioned above, a large-scale RCT evaluating the short version has previously been published [7]. The extended version used in the present study is composed of two modules. The first part includes the 12 sessions from the short version. The second part (sessions 13–24) is based on the first part with an enhanced individually tailored program for each participant. Each participant prepares for the second part by updating his or her goals together with the trainer. In the second part, each participant is also responsible for leading one group session, including activities and discussion. Activities outside the clinic are included in the second part, aiming to support the implementation and generalization of the acquired skills in real-life situations. Parents/caregivers participate in the first, mid and last session of both the first and the second part of the intervention (6 sessions in total), and in one individual meeting between the first and the second part to help formulate and update goals for the child/adolescent. **Online Resource 1** describes the content of each session in more detail.

### Standard care

Standard care included any ongoing support or intervention provided by regular health-care services (child psychiatry, paediatrics, habilitation centers, speech and language therapy). Information on the standard care for each participant was retrieved from their medical records. These included pharmacological treatments, occupational therapy, parental psychoeducation, counselling, and individual cognitive behaviour therapy (Table 1). All participants randomized to standard care were offered to take part in KONTAKT after completion of the trial period.

**Table 1:** Participants’ demographic and clinical characteristics at baseline

Participant characteristics	KONTAKT + standard care (*n* = 23)	Standard care (*n* = 27)	*P*
Age (years), mean (SD)	13.04 (2.58)	12.63 (2.83)	0.59
Males, *n* (%)	18 (78%)	17 (63%)	0.24
Language, *n* (%)			0.77
Swedish	21 (91%)	24 (89%)	
Other	2 (9%)	3 (11%)	
WISC-IV, Mean (SD)			
Full-scale IQ	98.57 (14.38)	95.59 (9.55)	0.39
Verbal IQ	102.78 (18.67)	97.11 (10.09)	0.18
Performance IQ	106.48 (11.46)	102.67 (14.68)	0.32
Working memory	86.43 (13.47)	91.78 (12.15)	0.15
Processing speed	93.70 (16.31)	90.85 (11.60)	0.48
ADOS, mean (SD)			
Total score	10.83 (3.45)	11.19 (3.13)	0.70
Communication	3.22 (1.31)	3.59 (1.72)	0.40
Reciprocal social interaction	7.61 (2.74)	7.59 (2.02)	0.98
ASD diagnosis, *n* (%)			0.21
Autistic disorder	1 (4%)	3 (11%)	
Asperger’s syndrome	14 (61%)	20 (74%)	
PDD-NOS	8 (35%)	4 (15%)	
Comorbidity, *n* (%)			
ADHD	16 (70%)	18 (67%)	0.83
Anxiety	6 (26%)	7 (26%)	0.99
Depression	8 (35%)	5 (19%)	0.19
Other	2 (9%)	4 (15%)	0.51
Pharmacological treatment, *n* (%)			
Stimulants	9 (39%)	15 (56%)	0.25
Sleep inducing	4 (17%)	3 (11%)	0.52
SSRI	7 (30%)	9 (33%)	0.83
Anti-histamine	8 (35%)	4 (15%)	0.10
Anti-psychotic	1 (4%)	2 (7%)	0.65
Anti-epileptic	0	0	–
Benzodiazepines	0	1 (4%)	0.35
Psychological treatment, *n* (%)			
CBT	0	1 (4%)	0.35
Counseling	1 (4%)	2 (7%)	0.65
Habilitation services, *n* (%)			
Parental psychoeducation	3 (13%)	2 (7%)	0.51
Other (e.g., cognitive aids, heavy weighted blankets)	4 (17%)	0	0.02
SRS-2 (parent), mean (SD)	91.13 (30.75)	88.96 (20.27)	0.77
SRS-2 (teacher), mean (SD)	69.14 (32.84)	73.16 (26.32)	0.67

*CBT* cognitive behavior therapy, *WISC-IV* Wechsler Intelligence Scale for Children 4th Edition, *ADOS* autism diagnostic observation schedule, *ASD* autism spectrum disorder, *PDD-NOS* pervasive developmental disorder not otherwise specified, *ADHD* attention-deficit hyperactivity disorder, *SSRI* selective serotonin reuptake inhibitor, *SRS-2* Social Responsiveness Scale 2nd Edition

### Outcome measures

The outcome measures included in the present study were selected based on their relevance for the clinical context in which the study was conducted.

#### Primary outcome measure

Social communication skills were measured using the SRS-2 [9]. The SRS-2 is a 65-item instrument measuring autistic-like traits across five domains—social awareness, social cognition, social communication, social motivation and autistic mannerisms. It has demonstrated high external validity, excellent internal consistency (Cronbach’s α = 0.97) and good test–retest reliability of 0.95–0.97 [23]. Both parent- and teacher-rated SRS-2 were included.

#### Secondary outcome measures

Adaptive skills were measured using the Adaptive Behavior Assessment System II (ABAS-II) [24]. The ABAS-II measures adaptive behavior using nine subscales composing three domains – cognitive skills, social skills, and practical skills at home, school and leisure time. It has been standardized and validated in both the US [24] and Sweden [25]. The Swedish standardization demonstrated excellent internal consistency (Cronbach’s α = 0.97–0.98) and acceptable correlations between parents’ and teachers’ ratings (*r* = 0.39). Symptom severity and clinical impression of everyday functioning were measured using the OSU Autism Clinical Global Impression scale (CGI) [26] and the Developmental Disabilities modification of the Children’s Global Assessment Scale (DD-CGAS) respectively [26]. Both instruments have recently been validated for Swedish conditions, showing acceptable inter-rater reliability for experienced raters (CGI-Aut, *r* = 0.72; DD-CGAS, *r* = 0.75) and sensitivity to clinical change [26]. Participant stress was measured using the Stress in Children questionnaire (SiC) [27]. The SiC is a 21-item instrument measuring stress using descriptions of physical and emotional symptoms of stress and has demonstrated good internal consistency (Cronbach’s α = 0.86). Parental stress was measured using the Perceived Stress Scale (PSS) [28]. The PSS is a 14-item instrument measuring stress related to everyday life. The Swedish translation has demonstrated good internal consistency (Cronbach’s α = 0.82) [29].

### Adverse events

Information about potential adverse events was extracted from a course evaluation completed at posttreatment and follow-up by the parents. The main purpose of the course evaluation was to identify aspects of the intervention that could be improved, and to collect information that would be useful for future implementation purposes. The questionnaire included free text questions about observed changes in the children and adolescent after entering the treatment and possible drawbacks of the training. An adverse event was defined as any reported occurrence, which is unfavorable for the participant, regardless of causality.

### Statistical analyses

Primary and secondary analyses were conducted according to intention-to-treat principles including all randomized participants for whom data were available at baseline. Mixed-effect linear modeling (random regression) [30] was used to provide unbiased treatment effects for primary and secondary outcomes. The model was specified using time (baseline, posttreatment, follow-up), treatment group (KONTAKT + standard care vs. standard care only), and the time by group interaction as fixed effects, with a random intercept for each participant. The results were presented as least squares means from the mixed-effect models. The treatment effect was expressed as the group difference in the change of least squares mean scores from pretreatment to posttreatment/follow-up. The slope of the regression line was compared between treatment groups. Effect sizes (ES) were estimated by dividing the group difference in the change of least squares mean scores from pretreatment to posttreatment/follow-up by the pooled standard deviation at pretreatment. Throughout the manuscript, positive effect sizes favor SSGT. Student’s *t* test and Pearson’s chi-squared test were used to determine if the two groups differed at baseline. The analyses were conducted using R software version 3.2 and IBM SPSS statistics version 24. The original plan was to include a comparable number of participants in the present trial and the simultaneously conducted RCT evaluating the shorter version of KONTAKT [7] (a total of approximately 144 participants receiving either the short or the long version of KONTAKT and an equivalent number of controls), but for practical reasons the majority of the clinics involved were only prepared to implement the 12-week version. This resulted in a smaller sample than expected for the present trial and limited power to detect small to medium effect sizes.

## Results

### Study flow and sample characteristics

**Fig. 1:**
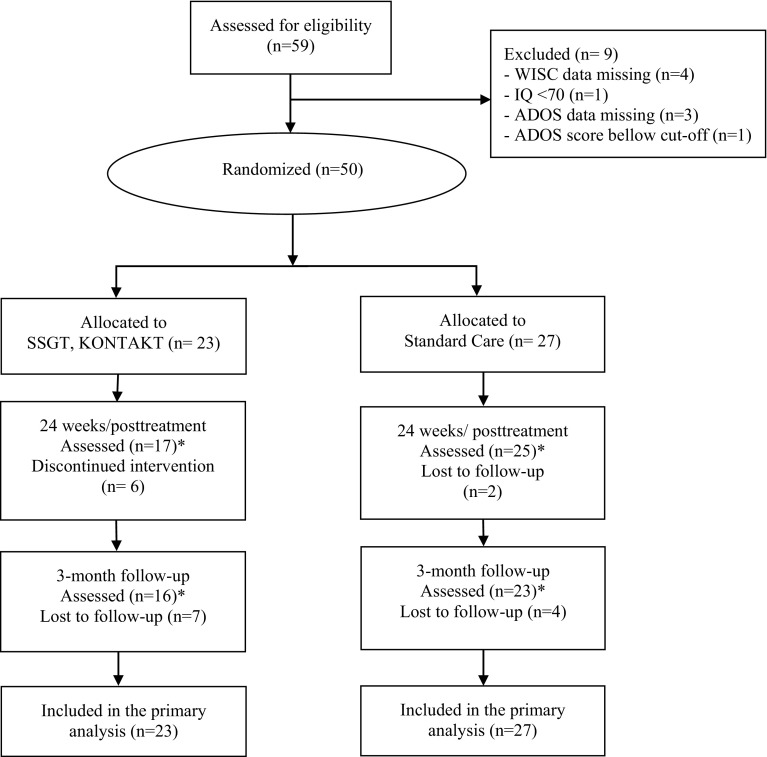
CONSORT Flow Diagram. *The number of participants assessed refer to parent-rated outcome measures

A total of 59 children and adolescents were assessed for eligibility. Out of these, *N* = 50 met the inclusion criteria and were randomly assigned to either 24 weeks of KONTAKT + standard care (*n* = 23) or standard care only (*n* = 27). Six participants discontinued the training and did not provide data posttreatment nor at follow-up. For the parent-rated measures complete posttreatment data were available for 17 (74%) participants in the KONTAKT group and 25 (93%) participants in the control group, while complete follow-up data were available for 16 (70%) participants in the KONTAKT group and 23 (85%) participants in the control group. Complete trainer-rated posttreatment assessments were available for 16 (70%) participants in the KONTAKT group and 25 (93%) participants in the control group, while complete follow-up assessments were available for 18 (78%) participants in the KONTAKT group and 26 (96%) participants in the control group. Complete teacher-rated posttreatment assessments were available for 9 (39%) participants in the KONTAKT group and 16 (59%) participants in the control group, while complete follow-up assessments were available for 12 (52%) participants in the KONTAKT group and 13 (48%) participants in the control group. Teacher-rated data should be interpreted with caution due to an unsatisfactory rate of complete data but are still reported for transparency reasons. Figure 1 shows participant flow from recruitment to follow-up.

Baseline demographic and clinical characteristics of the study participants are presented in Table 1. The groups did not differ significantly regarding diagnosis, age, gender, IQ, or comorbidity, or SRS-2 scores at baseline. The mean parent-rated SRS-2 at baseline was 91.13 (SD = 30.75) for the KONTAKT group and 88.96 (SD = 20.27) for the control group, indicating a social communication problem severity typical of ASD. The standard care provided did not differ significantly between the groups, with the exception that a significantly larger proportion of the KONTAKT group received habilitation services (e.g., assistive cognitive aids and heavy weighted blankets). The small group of participants in the KONTAKT group receiving such services (*n*  = 4) scored higher on SRS-2 pretreatment but had improved to the same extent as the rest of the KONTAKT group posttreatment and at follow-up. The demographic characteristic of the participating parents did not differ significantly (Table 2).

**Table 2:** Characteristics of the participating parents

Parental characteristics	KONTAKT + standard care (*n* = 23)	Standard care (*n* = 27)	*P*
Relationship to participant, *n* (%)			0.51
Mother	16 (70%)	21 (78%)	
Father	7 (30%)	6 (22%)	
Age (years), mean (SD)	47.43 (5.95)	48.52 (4.30)	0.46
Marital status, *n* (%)			0.30
Cohabiting	18 (78%)	17 (63%)	
Living apart	0	2 (7)	
Single parent	5 (22%)	8 (30%)	
No. of children, *n* (%)			0.29
1	2 (9%)	7 (26%)	
2	13 (57%)	13 (48%)	
3	8 (35%)	6 (22%)	
4+	0	1 (4%)	
Occupation of parent, *n* (%)			0.56
Full-time work	13 (57%)	15 (56%)	
Part-time work	7 (30%)	5 (19%)	
Student	0	1 (4%)	
Looking for work	0	0	
Sick leave	2 (9%)	2 (7%)	
Other	1 (4%)	4 (15%)	
Education (years), mean (SD)	15.63 (2.23)	14.59 (2.03)	0.22

### Primary outcomes

**Fig. 2:**
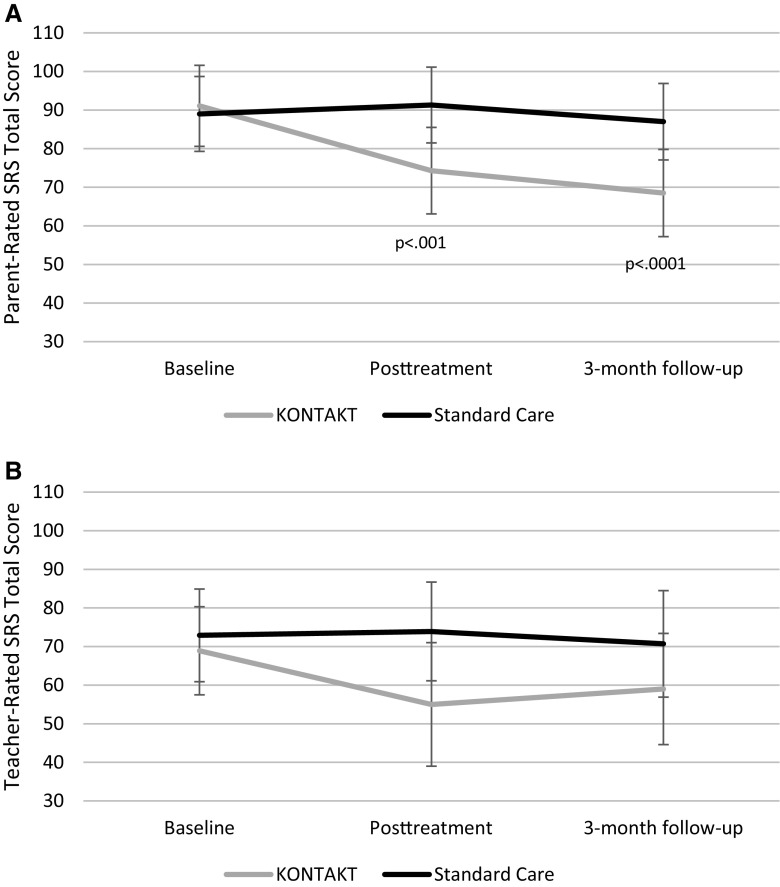
Least square means and 95% confidence intervals for parent- and teacher-rated Social Responsiveness Scale–Second Edition (SRS-2) scores at baseline, posttreatment and 3-month follow-up; significant time by group interactions are indicated by *p*-values

The group by time interaction indicated a significantly larger change in parent-rated SRS-2 total scores for the KONTAKT group posttreatment (treatment effect − 19.2; 95% CI, − 29.9 to − 8.5; *p* < .001; ES = 0.76) and at follow-up (treatment effect − 20.7; 95% CI − 31.7 to − 9.7; *p* < 0.0001; ES = 0.82) compared to the standard care group, indicating large effect sizes in favor of KONTAKT (Table 3; Fig. 2a). There was no significant group by time interaction for the teacher-rated SRS-2 total score posttreatment (Table 3; Fig. 2b).

**Table 3:** Primary outcome measures at baseline, week 24/posttreatment and 3-months follow-up

Measure	Mean (95% CI)^a^		Group difference in change score	*P*	Effect size (95% CI)^b^
	KONTAKT + standard care (*n* = 23)	Standard care (*n* = 27)			
SRS-2 (parent)					
Baseline	91.1 (80.7–101.6)	89.0 (79.3–98.6)			
Posttreatment	74.3 (63.1–85.5)	91.3 (81.5–101.1)	− 19.2 (− 29.9 to − 8.5)	0.001	0.76 (0.18–1.34)
Follow-up	68.5 (57.2–79.8)	87.0 (77.1–96.9)	− 20.7 (− 31.7 to − 9.7)	0.0001	0.82 (0.24–1.40)
SRS-2 (teacher)					
Baseline	68.9 (57.5–80.3)	72.9 (60.9–84.9)			
Posttreatment	55.0 (39.0–71.1)	73.9 (61.1–86.7)	− 14.9 (− 34.5 to 5)	0.15	0.50 (− 0.06 to 1.06)
Follow-up	59.0 (44.6–73.3)	70.7 (56.9–84.5)	− 7.7 (− 26.8 to 11.8)	0.45	0.26 (− 0.30 to 0.82)

*SRS-2* Social Responsiveness Scale 2nd Edition

^a^ Data are presented as raw mean scores at baseline and least squares mean values for week 24/posttreatment and 3 months follow-up with 95% CIs for each assessment point

^b^ Effect sizes were calculated by taking the difference in the least squares means at 24 week/posttreatment and follow-up dividing by the pooled standard deviation at baseline; positive effect sizes favor KONTAKT

### Secondary outcomes

There was a statistically significant difference between the groups in change scores on DD-CGAS posttreatment (treatment effect 5.2; 95% CI 0.2–10.2; *p* < 0.05; ES = 0.83), but the effect was no longer statistically significant at follow-up (treatment effect 1.8; 95% CI − 3.1 to 10.2; *p* = 0.48; ES = 0.29). There was no significant group by time interactions for the remaining secondary outcomes (Table 4).

**Table 4:** Secondary outcome measures at baseline, week 24/posttreatment and 3-months follow-up

Measure (rater)	Mean (95% CI)^a^		Group difference in change score	*P*	Effect size (95% CI)^b^
	KONTAKT + standard care (*n* = 23)	Standard care (*n* = 27)			
ABAS-II (parent)					
Baseline	388.0 (361.3–414.8)	376.0 (351.3–400.7)			
Posttreatment	411.6 (383.6–439.6)	386.5 (361.5–411.5)	13.1 (− 8.5 to 34.7)	0.25	0.19 (− 0.37 to 0.75)
Follow-up	427.2 (399.0–455.5)	399.1 (373.9–424.3)	16.1 (− 6 to 38.3)	0.16	0.24 (− 0.32 to 0.80)
ABAS-II (teacher)					
Baseline	361.1 (330.9–391.3)	339.0 (308.0–370.0)			
Posttreatment	386.2 (350.6–421.8)	344.2 (312.2–376.2)	19.9 (− 13.9 to 53.6)	0.27	0.26 (− 0.30 to 0.82)
Follow-up	379.3 (345.7–413.0)	349.6 (316.7–382.5)	7.6 (− 26.1 to 41.0)	0.67	0.10 (− 0.46 to 0.66)
DD-CGAS (trainer)					
Baseline	56.0 (52.9–59.1)	56.4 (53.5–59.2)			
Posttreatment	60.6 (57–64.2)	55.7 (52.8–58.7)	5.2 (0.2–10.2)	0.046	0.83 (0.25–1.41)
Follow-up	60 (56.6–63.4)	58.6 (55.7–61.4)	1.8 (− 3.1 to 6.6)	0.48	0.29 (− 0.27 to 0.85)
CGI-S (trainer)					
Baseline	4.3 (4.0–4.7)	4.5 (4.2–4.8)			
Posttreatment	4.1 (3.7–4.4)	4.4 (4.1–4.7)	− 0.2 (− 0.7 to 0.4)	0.56	0.31(− 0.25 to 0.87)
Follow-up	3.9 (3.6–4.3)	4.0 (3.7–4.3)	0.1 (− 0.5 to 0.6)	0.83	− 0.16 (− 0.72 to 0.40)
CiS (child self-report)					
Baseline	2.4 (2.2–2.6)	2.3 (2.2–2.5)			
Posttreatment	2.4 (2.2–2.7)	2.3 (2.1–2.5)	0.1 (− 0.1 to 0.3)	0.45	− 0.20 (− 0.76 to 0.36)
Follow-up	2.4 (2.1–2.6)	2.3 (2.1–2.5)	0.0 (− 0.2 to 0.2)	0.90	0.00 (− 0.56 to 0.56)
PSS (parental self-report)					
Baseline	26.3 (22.9–29.6)	25.9 (22.8–29.0)			
Posttreatment	20.1 (16.3–24)	23.4 (20.2–26.6)	− 3.6 (− 8.3 to 1.0)	0.14	0.46 (− 0.10 to 1.02)
Follow-up	19.1 (15.3–23)	21.6 (18.4–24.9)	− 2.8 (− 7.5 to 1.8)	0.24	0.36 (− 0.20 to 0.92)

*ABAS-II* adaptive behavior assessment system II, *CGI-S* OSU Autism Clinical Global Impression—Severity, *CiS* children in stress, *DD-CGAS*, developmental disabilities modification of the Children’s Global Assessment Scale, *PSS* Perceived Stress Scale

^a^ Data are presented as raw mean scores at baseline and least squares mean values for week 12/posttreatment and 3 months follow-up with 95% CIs for each assessment point

^b^ Effect sizes were calculated by taking the difference in the least squares means at 24 week/posttreatment and follow-up dividing by the pooled standard deviation at baseline; positive effect sizes favor KONTAKT

### Adverse events

Parent-reported course evaluations were available for 16 of the 23 participants (70%) in the KONTAKT group. Occurrences defined as adverse events were reported by a total of seven parents and included the following: treatment non-response (*n* = 3); missed time in school (*n* = 1); social withdrawal and depressed mood as a possible consequence of increased awareness of challenges related to ASD (*n* = 1); feeling uncomfortable/annoyed with the other group members (*n* = 2). We found no indications that the subgroup experiencing an adverse event deteriorated on the outcome measures. The parents reporting such an event still affirmed that they would recommend the intervention to others, with the exception of one parent who was uncertain.

## Discussion

The present study examined the effects of 24 weekly sessions of SSGT KONTAKT compared with standard care for children and adolescents in the normative IQ range with ASD. The study was conducted in real-world clinical settings with a sample of children and adolescents typical of clients with ASD referred to child and adolescent psychiatry. The positive effect on parent-rated social communication and other autistic trait related social skills was large, both posttreatment and 3 months after completion. The point estimate suggested a substantially larger effect than what has been reported for shorter SSGT programs. Thus, this study endorses the potential benefit of prolonged training previously voiced by young individuals undertaking shorter social skills training programs [31].

Parallel to the present study, a large-scale RCT evaluating the 12-week version of the KONTAKT program was conducted by the same research group [7]. The procedures of the two studies were largely identical: they were conducted during the same time period within the same clinical settings, used staff with comparable training, recruited samples in the same manner, and included samples with highly similar characteristics. While the evaluation of the shorter version of KONTAKT led us to conclude that the intervention is feasible and safe in routine care, the estimated effects were modest and inconsistent. Three months after completion the participants undertaking the 12-week training on average had improved 11 points from baseline on the parent-rated SRS. As a comparison, those participating in the 24-week training had on average improved 23 points 3 months after completion. Thus, the average change score was more than twice the magnitude for the extended version. A few additional RCTs evaluating SSGT programs have included parent-rated SRS as an outcome measure, allowing for a rough comparison of the change scores in the treatment groups. A German multi-center study evaluating a 14-week SSGT-program derived from KONTAKT, reported a mean reduction of 15 points on the parent-rated SRS in the treatment group 3 months after the intervention [8]. Two studies of an intensive 5-week program in 7–12-year-olds yielded an average improvement of less than 10 points [32, 33]. The same was true for a small study of a 15-week social skills program based on cognitive behavioral principles [34], and a pilot study comparing two different 4-week social skills interventions in a limited sample [35]. A somewhat larger change (an average improvement of 14 points) was observed in a trial evaluating a multimodal anxiety and social skills intervention for adolescents [36]. Finally, two separate RCTs have evaluated the 14-week Program for the Education and Enrichment of Relational Skills (PEERS) in children and adolescents aged 11–18 years [37, 38]. The treatment group yielded an average improvement of 10 points in one of these studies [38] and 22 points in the other [37]. The latter was the only previous study we could find with results that were on a par with ours.

Group differences in teacher-rated SRS and secondary outcome measures did not reach statistical significance in the present study, with the exception of trainer-rated DD-CGAS posttreatment. However, the estimated improvement on these measures was comparable to those reported for the shorter version of KONTAKT. The previous evaluation of the shorter version indicated statistically significant effects in favor of the intervention on several of the secondary measures [7], which highlights the limited precision of the present study and stresses the need for larger samples to better estimate the true effect of long-term training. The results on adverse effects in the present trial were comparable to those previously reported for the shorter version [7] and underscore the importance of identifying subgroups that are unlikely to benefit from this particular intervention and monitor closely the group dynamics. Some adverse reactions (e.g., related to intragroup dynamics and gained insights) might be avoided by minor adjustments to the intervention. Overall, our data on adverse events serve to illustrate the value of such information, which has not been routinely monitored and reported in psychosocial intervention research [40].

While our preliminary results suggest that there are benefits of extended training, there are also costs such as personnel and other resources as well as time and effort on the part of the client. Notably, a majority of clinics involved in the evaluation of KONTAKT were not prepared to implement the longer version for the present trial. To use available resources effectively, service providers must have access to information that enables them to make informed decisions about the optimal length of the intervention in terms of both costs and benefits. This would require not only estimates of higher precision than the ones we provide here, but also a more detailed “dose–response” characterization. This is a key component of the development and evaluation of pharmaceutical products, where any given dose provides a mixture of desirable and undesirable effects [39]. Dose–response curves for both effectiveness and unwanted effects can help to clarify if a dose is beyond further beneficial effects, or if the risk of undesirable effects increase or decrease with the length or intensity of the intervention. So far, this has not been an integral part of the development of psychosocial interventions. While we believe that the “dose–response” analogue could be useful also in this research field, inherent differences between social skills training and pharmacological treatment must also be considered. A “higher dose” of social skills training, for instance, is not necessarily just more of the same. There is a natural progress in the acquisition of a new skill, from practicing the skill in a controlled and safe environment to applying the skill in everyday life. Longer training will inevitably allow for more applied and individualized training, once the basic skills are acquired. The more favorable outcome of the extended version of KONTAKT suggested by the present study might, therefore, partly be explained by qualitative rather than quantitative differences between the two versions of the intervention.

Future research should attempt to clarify further the mechanisms involved in SSGT and its effects. While longer duration allows for overlearning and consolidation of acquired skills, other aspects of the intervention might be just as important. It is, for instance, not clear if more intense treatments over shorter periods of time would have the same benefits [32, 33]. Further, the role of parent involvement in maintaining the training and the acquired skills is an important focus of future investigation. It is, for instance, possible that the quality of parental involvement improves over time, which would favor longer interventions. To move beyond the current standard of social skills training, including the KONTAKT program, future research also needs to identify characteristics of the training where there still is potential for improvement. Modern approaches to skills acquisition in general (e.g., deliberate practice) would point to aspects such as high-quality feedback, methods to maintain focused practice and motivation, and strategies to move past plateaus [11]. Another possibility would be to find ways to effectively shape the individual’s mental representation of social situations held in the long-term memory, making it possible to respond quickly and adequately in such situation despite the inherent limitations of the short-term memory [41]. These are not explicit components of the KONTAKT program in its current form.

### Limitations

The present study has some limitations that deserve to be addressed explicitly. First, the blinded teacher ratings were deemed unreliable due to a large amount of missing data. Thus, our conclusions are mainly based on unblinded parent ratings, and we cannot rule out that their assessments were biased. Teachers, on the other hand, reported that they sometimes did not have sufficient insight to be qualified to assess the abilities of individual students. This is unfortunate since gains observed by teachers would suggest that the intervention effects have generalized outside the home environment. Many studies in this field rely solely on unblinded measures which underscores the need for blinded assessment tools that are both valid and obtainable [42, 43]. Second, no head-to-head comparison with a shorter SSGT program was included in the trial. We are fairly confident that the indirect comparison with the short version of KONTAKT presented in the discussion is valid, given the almost identical procedures used in the two trials. A major difference between the trials was that the present trial was conducted at only two of the 13 clinics involved in the evaluation of the short version, but we find it unlikely that this discrepancy had a major impact on the outcome. Third, as mentioned above, the relatively small sample size resulted in estimates of limited precision. Thus, the point estimates reported here must be interpreted with caution. In addition, the limited sample did not permit analyses of moderators and mediators of the training effect. The previous evaluation of KONTAKT suggested that the effect partly was moderated by age and sex [7]. It would be of paramount interest to clarify if these differences also remain when the training period is prolonged, or whether different moderators apply. Finally, some of the interventions received as part of standard care (e.g., CBT and counseling) might have content that partly overlaps with KONTAKT. This might have led to an underestimation of the true effect of KONTAKT, although the low number of individuals receiving such interventions would suggest that any such effect was minimal. Similarly, four participants in the KONTAKT group (and none in the control group) received habilitation services such as heavy weighted blankets and cognitive aids for structuring one’s daily living. We have no reason to believe that this had a noteworthy impact on the results and found no such indications.

## Conclusion

The present study suggests that a long-term SSGT program can result in larger gains in social skills than has previously been reported for shorter SSGT programs. The gains were maintained 3 months after completion of the training. While these preliminary results should be interpreted with caution, they hold promise that continued efforts to fine-tune the content and form of social skills training programs eventually could enable service providers to use their resources more effectively and help young people with ASD to reach their full potential.

## Electronic supplementary material

Below is the link to the electronic supplementary material.

Supplementary material 1 (DOCX 19 KB)
