# Supplementary material for: Long-term social skills group training for children and adolescents with autism spectrum disorder: a randomized controlled trial
Source: Eur Child Adolesc Psychiatry. 2018 May 10;28(2):189–201. doi: 10.1007/s00787-018-1161-9 (PMC6510850; doi:10.1007/s00787-018-1161-9)
Supplement: Supplementary file 1 — Supplementary material 1 (DOCX 19 KB) [file 787_2018_1161_MOESM1_ESM.docx]

**Online resource 1**

| **Overview of the session structure and contents of the long version of the Social Skills Group Training KONTAKT** | | | | | | |
| --- | --- | --- | --- | --- | --- | --- |
| *First part (session 1-12)* | | | | | | |
| **Session** | **Purpose** | **Group exercise(s)** | | **Topic(s) for group discussion** | | **Homework assignment** |
| 1. | Introduction to the group and information about the intervention (parents participate) | Participants give a short presentation of themselves, mentioning one of their strengths | | Confidentiality; introduction to the workbook | | Formulate an individual goal |
| 2 | Getting to know the other participants and agreeing upon common rules | Spin the bottle; Tell something about yourself | | Establishing group rules, learning about each other’s interests and abilities | | Set intermediate goals and identify the supporting network. Describe a situation related to the goals (functional analysis) |
| 3 | Psychoeducation about ASD with focus on strengths; recognize facial expressions | FEFA^a^; Spin the bottle; Your favorite pastime | | What do we know about ASD? Strengths and strategies to overcome difficulties? | | Functional analysis of a situation that was difficult to cope with (e.g., a situation where you got angry) |
| 4 | Basic emotions, cooperation, and nonverbal communication | What has changed?; The blinking game | | How do I know if someone is happy, sad, angry or neutral? | | Functional analysis of a situation relating to the goals, with focus on short-term vs. long-term consequences |
| 5 | Complex emotions, body language, cooperation and nonverbal communication | FEFA; The fruit basket; Role play | | How are emotions expressed through gestures and facial expression interpreted? | | Functional analysis of a situation where the participant used an alternative way to handle a difficult situation |
| 6 | Describe the intentions of others and their body language (parents participate) | Spin the bottle; Pantomime | | - | | Functional analysis of a situation involving nonverbal communication |
| 7 | Misunderstandings, mixed emotions, and nonverbal communication | The blinking game; What has changed?; Role play | | Misunderstandings; understanding irony, sarcasm, and white lies | | Functional analysis of a situation where the participant started a conversation |
| 8 | Feelings of alienation and problem-solving | FEFA; Spin the bottle; Scavenger hunt | | How to handle unfamiliar social situations | | Functional analysis of a situation where the participant felt lonely or excluded |
| 9 | Being with people you don’t know | Pantomime; Talk to someone you don’t know | | Loneliness and being bullied | | Functional analysis of a situation where the participant made an appointment with someone who cancelled |
| 10 | Getting in touch with your friends | Role play; Chinese whispers; Spin the bottle | | How can I arrange a playdate/meeting with a friend? What do I do if they cancel? | | Functional analysis of a situation where the participant had a conversation with a stranger or somebody he/she did not know well |
| 11 | Reaching out to people you don’t know | A common activity (e.g., baking or going to a café) | | To communicate with a stranger, for example when you go shopping | | Evaluation of KONTAKT. How to maintain and improve acquired skills? |
| 12 | Evaluation and future improvements (patents participate) | - | | Evaluation of KONTAKT and lessons learned. Strategies for further improvement | | - |
| *Second part (session 13-24)* | | | | | | |
|  | The second part aims to support implementation and generalization of the acquired skills in real-life situations, and has a more flexible structure than the first. Each participant prepares for the second part by updating their goals together with the KONTAKT trainer. Homework assignments are tailored for each participant, and each participant plans and leads one of the concluding sessions together with the trainers. To allow for each participant to lead their own session, the number of sessions with group discussions based on the participants’ individual goals can vary depending on the group size. If two or more participants have similar individual goals, these might be discussed during the same session. | | | | | |
|  | **Purpose** | **Group exercise(s)** | **Topic(s) for group discussion** | | **Homework assignment** | |
| 13 | Introduction (parents participate) | Decided by the trainer (e.g., What has changed?) | Focus on the content and structure of the upcoming sessions and group discussion based on the participants’ individual goals | | Formulate an individual goal | |
| 14 | Generalization of skills | Decided by the trainer (e.g., baking) | Based on the participants’ individual goals | | Individually tailored assignment | |
| 15 | Generalization of skills | Decided by the trainer (e.g., role play) | Based on the participants’ individual goals | | Individually tailored assignment | |
| 16 | Generalization of skills | Decided by the trainer (e.g., film about non-verbal communication) | Based on the participants’ individual goals | | Preparation for the excursion and Individually tailored assignment | |
| 17 | Generalization of skills | Going out together (e.g., to a café) | What to think about when you, for instance, are in a café? | | Functional analysis of the excursion | |
| 18 | Evaluation of the excursion (parents participate) | Decided by the trainer (e.g., scavenger hunt) | Based on the participants’ individual goals | | Individually tailored assignment | |
| 19 | Generalization of skills | Decided by the participant responsible for the session | Decided by the participant responsible for the session | | Individually tailored assignment | |
| 20 | Generalization of skills | Decided by the participant responsible for the session | Decided by the participant responsible for the session | | Individually tailored assignment | |
| 21 | Generalization of skills | Decided by the participant responsible for the session | Decided by the participant responsible for the session | | Individually tailored assignment | |
| 22 | Generalization of skills | Decided by the participant responsible for the session | Decided by the participant responsible for the session | | Individually tailored assignment | |
| 23 | Generalization of skills | Decided by the participant responsible for the session | Decided by the participant responsible for the session | | Evaluation of KONTAKT and strategies for maintenance and further improvement. Write down three positive things about each of the other participants | |
| 24 | Evaluation  (parents participate) | Decided by the whole group | Evaluation of the acquired skills and focus on strategies for maintenance and further improvement | |  | |
| ^a^ A computer-based program to teach the recognition of facial affect (Bölte S, Feineis-Matthews S, Leber S, Dierks T, Hubl D, Poustka F (2002). The development and evaluation of a computer-based program to test and to teach the recognition of facial affect. Int J Circumpolar Health 61:61-68) | | | | | | |
